# Recruitment-to-inflation Ratio Assessed through Sequential End-expiratory Lung Volume Measurement in Acute Respiratory Distress Syndrome

**DOI:** 10.1097/ALN.0000000000004716

**Published:** 2023-07-31

**Authors:** Domenico Luca Grieco, Gabriele Pintaudi, Filippo Bongiovanni, Gian Marco Anzellotti, Luca Salvatore Menga, Melania Cesarano, Antonio M. Dell’Anna, Tommaso Rosá, Luca Delle Cese, Giuseppe Bello, Valentina Giammatteo, Veronica Gennenzi, Eloisa S. Tanzarella, Salvatore L. Cutuli, Gennaro De Pascale, Andrea De Gaetano, Salvatore M. Maggiore, Massimo Antonelli

**Affiliations:** 1 Department of Anesthesiology and Intensive Care Medicine, Catholic University of the Sacred Heart, Rome, Italy; Anesthesia, Emergency and Intensive Care Medicine, Fondazione Policlinico Universitario A. Gemelli IRCCS, Rome, Italy.; 2 Department of Anesthesiology and Intensive Care Medicine, Catholic University of the Sacred Heart, Rome, Italy; Anesthesia, Emergency and Intensive Care Medicine, Fondazione Policlinico Universitario A. Gemelli IRCCS, Rome, Italy.; 3 Department of Anesthesiology and Intensive Care Medicine, Catholic University of the Sacred Heart, Rome, Italy; Anesthesia, Emergency and Intensive Care Medicine, Fondazione Policlinico Universitario A. Gemelli IRCCS, Rome, Italy.; 4 Department of Medical, Oral and Biotechnological Sciences, School of Medicine and Health Sciences, Section of Anesthesia, Analgesia, Perioperative and Intensive Care, SS, Annunziata Hospital, Gabriele d’Annunzio University of Chieti-Pescara, Chieti, Italy.; 5 Department of Anesthesiology and Intensive Care Medicine, Catholic University of the Sacred Heart, Rome, Italy; Anesthesia, Emergency and Intensive Care Medicine, Fondazione Policlinico Universitario A. Gemelli IRCCS, Rome, Italy.; 6 Department of Anesthesiology and Intensive Care Medicine, Catholic University of the Sacred Heart, Rome, Italy; Anesthesia, Emergency and Intensive Care Medicine, Fondazione Policlinico Universitario A. Gemelli IRCCS, Rome, Italy.; 7 Department of Anesthesiology and Intensive Care Medicine, Catholic University of the Sacred Heart, Rome, Italy; Anesthesia, Emergency and Intensive Care Medicine, Fondazione Policlinico Universitario A. Gemelli IRCCS, Rome, Italy.; 8 Department of Anesthesiology and Intensive Care Medicine, Catholic University of the Sacred Heart, Rome, Italy; Anesthesia, Emergency and Intensive Care Medicine, Fondazione Policlinico Universitario A. Gemelli IRCCS, Rome, Italy.; 9 Department of Anesthesiology and Intensive Care Medicine, Catholic University of the Sacred Heart, Rome, Italy; Anesthesia, Emergency and Intensive Care Medicine, Fondazione Policlinico Universitario A. Gemelli IRCCS, Rome, Italy.; 10 Department of Anesthesiology and Intensive Care Medicine, Catholic University of the Sacred Heart, Rome, Italy; Anesthesia, Emergency and Intensive Care Medicine, Fondazione Policlinico Universitario A. Gemelli IRCCS, Rome, Italy.; 11 Department of Anesthesiology and Intensive Care Medicine, Catholic University of the Sacred Heart, Rome, Italy; Anesthesia, Emergency and Intensive Care Medicine, Fondazione Policlinico Universitario A. Gemelli IRCCS, Rome, Italy.; 12 Department of Anesthesiology and Intensive Care Medicine, Catholic University of the Sacred Heart, Rome, Italy; Anesthesia, Emergency and Intensive Care Medicine, Fondazione Policlinico Universitario A. Gemelli IRCCS, Rome, Italy.; 13 Department of Anesthesiology and Intensive Care Medicine, Catholic University of the Sacred Heart, Rome, Italy; Anesthesia, Emergency and Intensive Care Medicine, Fondazione Policlinico Universitario A. Gemelli IRCCS, Rome, Italy.; 14 Department of Anesthesiology and Intensive Care Medicine, Catholic University of the Sacred Heart, Rome, Italy; Anesthesia, Emergency and Intensive Care Medicine, Fondazione Policlinico Universitario A. Gemelli IRCCS, Rome, Italy.; 15 Department of Anesthesiology and Intensive Care Medicine, Catholic University of the Sacred Heart, Rome, Italy; Anesthesia, Emergency and Intensive Care Medicine, Fondazione Policlinico Universitario A. Gemelli IRCCS, Rome, Italy.; 16 Consiglio Nazionale delle Ricerche, IRIB Istituto per la Ricerca e l’Innovazione Biomedica, Palermo, Italy; IASI Istituto per l’Analisi dei Sistemi ed Informatica, Rome, Italy; Department of Biomatics, Óbuda University, Budapest, Hungary.; 17 Department of Medical, Oral and Biotechnological Sciences, School of Medicine and Health Sciences, Section of Anesthesia, Analgesia, Perioperative and Intensive Care, SS, Annunziata Hospital, Gabriele d’Annunzio University of Chieti-Pescara, Chieti, Italy.; 18 Department of Anesthesiology and Intensive Care Medicine, Catholic University of the Sacred Heart, Rome, Italy; Anesthesia, Emergency and Intensive Care Medicine, Fondazione Policlinico Universitario A. Gemelli IRCCS, Rome, Italy.

## Abstract

**Background:**

Positive end-expiratory pressure (PEEP) benefits in acute respiratory distress syndrome are driven by lung dynamic strain reduction. This depends on the variable extent of alveolar recruitment. The recruitment-to-inflation ratio estimates recruitability across a 10–cm H_2_O PEEP range through a simplified maneuver. Whether recruitability is uniform or not across this range is unknown. The hypotheses of this study are that the recruitment-to-inflation ratio represents an accurate estimate of PEEP-induced changes in dynamic strain, but may show nonuniform behavior across the conventionally tested PEEP range (15 to 5 cm H_2_O).

**Methods:**

Twenty patients with moderate-to-severe COVID-19 acute respiratory distress syndrome underwent a decremental PEEP trial (PEEP 15 to 13 to 10 to 8 to 5 cm H_2_O). Respiratory mechanics and end-expiratory lung volume by nitrogen dilution were measured the end of each step. Gas exchange, recruited volume, recruitment-to-inflation ratio, and changes in dynamic, static, and total strain were computed between 15 and 5 cm H_2_O (global recruitment-to-inflation ratio) and within narrower PEEP ranges (granular recruitment-to-inflation ratio).

**Results:**

Between 15 and 5 cm H_2_O, median [interquartile range] global recruitment-to-inflation ratio was 1.27 [0.40 to 1.69] and displayed a linear correlation with PEEP-induced dynamic strain reduction (r = –0.94; *P* < 0.001). Intraindividual recruitment-to-inflation ratio variability within the narrower ranges was high (85% [70 to 109]). The relationship between granular recruitment-to-inflation ratio and PEEP was mathematically described by a nonlinear, quadratic equation (*R*^2^ = 0.96). Granular recruitment-to-inflation ratio across the narrower PEEP ranges itself had a linear correlation with PEEP-induced reduction in dynamic strain (r = –0.89; *P* < 0.001).

**Conclusions:**

Both global and granular recruitment-to-inflation ratio accurately estimate PEEP-induced changes in lung dynamic strain. However, the effect of 10 cm H_2_O of PEEP on lung strain may be nonuniform. Granular recruitment-to-inflation ratio assessment within narrower PEEP ranges guided by end-expiratory lung volume measurement may aid more precise PEEP selection, especially when the recruitment-to-inflation ratio obtained with the simplified maneuver between PEEP 15 and 5 cm H_2_O yields intermediate values that are difficult to interpret for a proper choice between a high and low PEEP strategy.

## Abstract 2

In a cohort of patients with moderate to severe COVID-19 acute respiratory distress syndrome, the authors determined respiratory mechanics and end-expiratory lung volume by nitrogen dilution over a 10–cm H_2_O decremental five-step positive end-expiratory pressure (PEEP) trial, and measured the recruitment-to-inflation ratio both globally (10–cm H_2_O PEEP range) and granularly (narrower PEEP ranges). The global effect displayed a linear correlation of both global and granular recruitment-to-inflation ratio with dynamic strain reduction. However, granular recruitment-to-inflation ratio showed high intraindividual variability, suggesting nonuniform recruitability across a 10–cm H_2_O PEEP range. A nonlinear quadratic relationship was noted between granular recruitment-to-inflation ratio and PEEP. These pilot data suggest that the recruitment-to-inflation ratio measured across a 10–cm H_2_O range may be of value clinically for setting PEEP to minimize lung dynamic strain. Granular recruitment-to-inflation ratio assessment may help more precisely assess PEEP effects, with relevant clinical implications for PEEP setting in patients who display a global recruitment-to-inflation ratio close to intermediate values.

## Visual Abstract:

**Figure:**
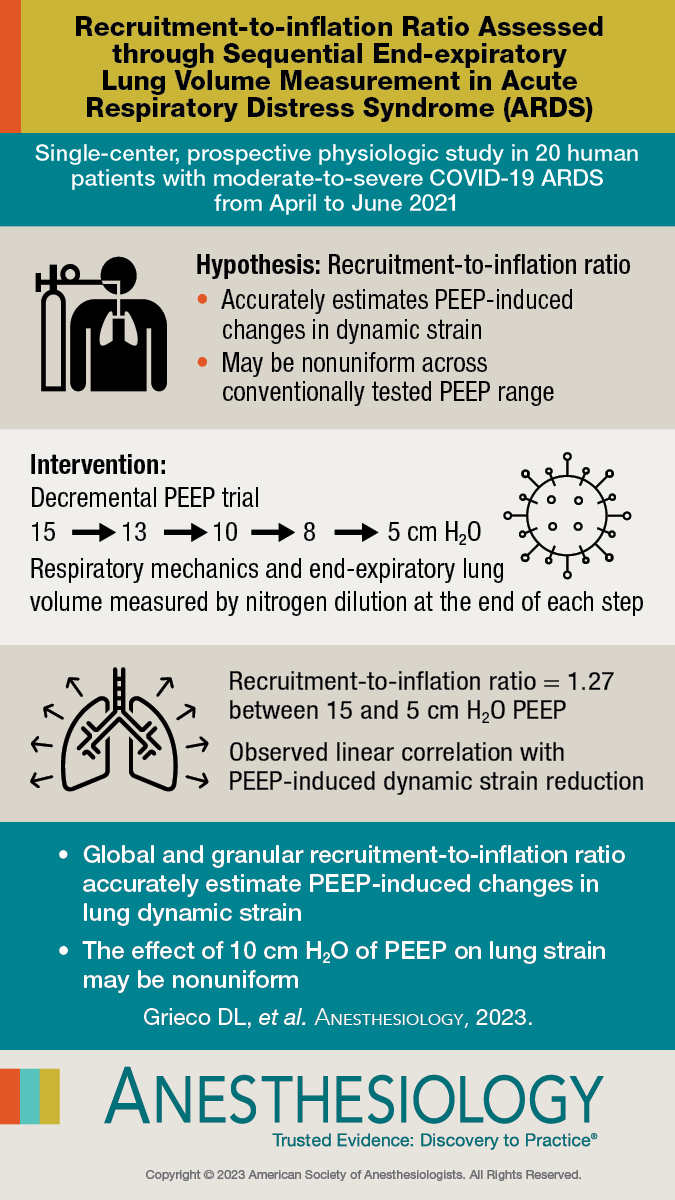

**Editor’s Perspective**

**What We Already Know about This Topic**

The ability to determine the optimal positive end-expiratory pressure (PEEP) for patients with acute respiratory distress syndrome may have important clinical ramifications.

The optimal PEEP in such patients aims at reducing lung dynamic strain while avoiding baby lung overinflation. The balance between these two effects depends on the variable extent of lung recruitment.

The recruitment-to-inflation ratio over a 10–cm H_2_O PEEP range has recently been proposed as a simplified method to bedside estimate lung recruitment, but it is undemonstrated if the recruitment-to-inflation ratio accurately reflects PEEP effects on lung strain.

Whether the recruitment-to-inflation ratio is uniform or not across a 10–cm H_2_O PEEP range is unknown.

**What This Article Tells Us That Is New**

In a cohort of patients with moderate to severe COVID-19 acute respiratory distress syndrome, the authors determined respiratory mechanics and end-expiratory lung volume by nitrogen dilution over a 10–cm H_2_O decremental five-step PEEP trial, and measured the recruitment-to-inflation ratio both globally (10–cm H_2_O PEEP range) and granularly (narrower PEEP ranges).

The global effect displayed a linear correlation of both global and granular recruitment-to-inflation ratio with dynamic strain reduction. However, granular recruitment-to-inflation ratio showed high intraindividual variability, suggesting nonuniform recruitability across a 10–cm H_2_O PEEP range.

A nonlinear quadratic relationship was noted between granular recruitment-to-inflation ratio and PEEP.

These pilot data suggest that the recruitment-to-inflation ratio measured across a 10–cm H_2_O range may be of value clinically for setting PEEP to minimize lung dynamic strain. Granular recruitment-to-inflation ratio assessment may help more precisely assess PEEP effects, with relevant clinical implications for PEEP setting in patients who display a global recruitment-to-inflation ratio close to intermediate values.

Mechanical ventilation represents the mainstay of supportive therapy in the acute respiratory distress syndrome (ARDS), aiming at maintaining gas exchange while limiting the occurrence of ventilator-induced lung injury.^1^ Well-established strategies recommended to avoid ventilator-induced lung injury include the use of low tidal volumes and the extensive use of prone position.^2^ However, the role of positive end-expiratory pressure (PEEP) remains uncertain, and there is no conclusive evidence to support the setting of an all-encompassing higher *versus* lower PEEP strategy.^3^

A high interindividual heterogeneity in the potential for lung recruitment with PEEP has been described.^4–6^ The application of PEEP generates overinflation in the already open tissue, while it may reduce the harmful effects of mechanical ventilation only when it yields significant recruitment of previously collapsed alveolar units. PEEP-induced alveolar recruitment reduces dynamic strain, defined as the ratio of tidal volume to aerated lung size.^7–10^ According to these considerations, the development of bedside strategies to assess the individual potential for lung recruitment appears warranted.^11^

Measurement of end-expiratory lung volume through the nitrogen dilution technique looks promising in this context^12,13^: it allows the direct measurement of aerated lung size at different PEEP levels, thereby enabling computation of alveolar recruitment, and static and dynamic strain.^14,15^ The recruitment-to-inflation ratio has been recently proposed to provide an estimate of the balance between PEEP-induced alveolar recruitment and PEEP-induced inflation for a given “baby lung” size. This index normalizes the compliance of the recruited lung (defined as the absolute recruited lung volume between two PEEP levels divided by the change in PEEP, thus obtaining the effective recruited volume per cm H_2_O of PEEP difference) to the respiratory system compliance at the lower PEEP level, which is an estimate of actual “baby lung” size.^16^

The recruitment-to-inflation ratio is assessed through a simplified maneuver and estimates the potential for lung recruitment within a 10–cm H_2_O PEEP range, essentially dichotomizing patients with high or low potential for lung recruitment.^6,16–18^ It is unknown if recruitability is uniform or not across this range. This may be especially relevant for intermediate recruitment-to-inflation ratio values (*i.e.*, 0.5 to 1), which may be difficult to interpret at the bedside to choose between high and low PEEP.^19^ It is also undemonstrated whether the recruitment-to-inflation ratio accurately reflects the impact of applied PEEP on lung dynamic strain. Importantly, the beneficial effects of PEEP on lung protection critically depend on its capability to reduce dynamic strain.^20^

We report the results of a physiologic study conducted in moderate-to-severe COVID-19 ARDS to determine the behavior of recruitment-to-inflation ratio across the conventionally tested 15– to 5–cm H_2_O PEEP range, assessed by sequential measurement of lung volumes during a decremental PEEP trial. Also, we wanted to test whether the recruitment-to-inflation ratio accurately reflects changes in lung strain produced by PEEP. The hypotheses of this study are that the recruitment-to-inflation ratio represents an accurate estimate of the change in dynamic strain produced by PEEP, but may show a nonuniform behavior across the conventionally tested PEEP range (15 to 5 cm H_2_O).

## Materials and Methods

This prospective, physiologic study was conducted in the intensive care unit (ICU) of a tertiary care university hospital between April and June 2021. Approval was obtained by local institutional review board, Comitato Etico Policlinico Gemelli. Written informed consent was obtained by patients’ legal representatives according to committee recommendations (ID UCSC915920/20). Withdrawal of consent to study participation or data analysis by patients or their legal representatives was allowed at any time during the study.

### Patients

Consecutive patients admitted to the ICU between April and July 2021 and fulfilling moderate-to-severe ARDS criteria^21,22^ were enrolled within 24 h from endotracheal intubation. Exclusion criteria were age younger than 18 yr, pregnancy, signs of barotrauma, severe hemodynamic instability (defined as norepinephrine dose greater than 0.5 mcg · kg^-1^ · min^-1^ and/or blood lactate greater than 5 mmol · l^-1^), and pre-existing decompensated heart failure (New York Heart Association class 3 to 4 and/or documented left ventricular ejection fraction less than 35%). Due to the ongoing pandemic, all enrolled patients happened to have COVID-19–associated respiratory failure.

### Procedures and Measurements

All patients were in the semirecumbent position (30° to 45° head-of-the-bed elevation) and sedated according to standard practice. Neuromuscular blockade was accomplished by cisatracurium continuous infusion at the dose of 35 mg · h^-1^. The patient was connected to a ventilator equipped with an end-expiratory lung volume measurement module (Carescape R860, GE Healthcare, USA) through a standard circuit and a heat and moisture exchanger. Ventilation settings were as follows: volume-controlled mode with tidal volume (V_T_), 6 ml · kg^-1^ of predicted body weight; inspiratory flow, 60 l · min^-1^; inspiratory pause, 0.3 s; and respiratory rate titrated to obtain pH between 7.35 and 7.45 with fractional inspired oxygen tension (Fio_2_) titrated to an oxygen saturation measured by pulse oximetry between 90 and 96%. Before starting the study measurements, a low-flow (5 l · min^-1^) inflation after prolonged exhalation (8 s) at zero PEEP was performed to assess for the presence of intrinsic PEEP and airway closure.^23,24^ Patients who displayed intrinsic PEEP greater than 2 cm H_2_O and/or airway closure with airway opening pressure greater than 5 cm H_2_O were not considered eligible for the study.

**Fig. 1.**
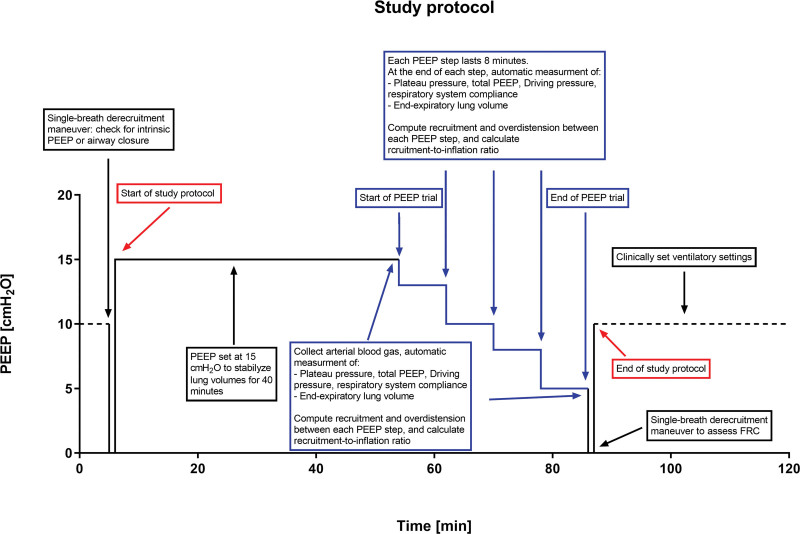
Flow chart of the study protocol. After assessment of eligibility, a single-breath derecruitment maneuver, followed by slow inflation, was performed to assess the presence of airway closure. Patients presenting with airway closure were excluded. After enrollment, each patient was ventilated at positive end-expiratory pressure (PEEP) 15 cm H_2_O for 40 min to stabilize lung volumes. Before the start of the PEEP trial, an arterial blood gas sample was collected, and respiratory mechanics were assessed by the ventilator. Afterward, a five-step decremental PEEP trial (PEEP 15 to 13 to 10 to 8 to 5 cm H_2_O) was conducted with the dedicated software of the ventilator. Each PEEP step lasted 8 min. At each step, the ventilator automatically measured and collected respiratory mechanics (plateau pressure, total PEEP, driving pressure, respiratory system compliance, and end-expiratory lung volume). At the end of the PEEP trial, blood gases were analyzed, and a single-breath derecruitment maneuver was performed to assess functional residual capacity (FRC). After the end of the study, ventilation was resumed as clinically indicated by the attending physician.

Study procedures are summarized in figure 1.^25^ PEEP was initially set at 15 cm H_2_O (provided that plateau and driving pressures did not exceed 30 cm H_2_O and 15 cm H_2_O, respectively) for 40 min to stabilize lung volumes; afterward, respiratory mechanics were assessed through standard occlusions, and arterial blood gases were analyzed. Subsequently, an automated five-step decremental PEEP trial (PEEP 15 to 13 to 10 to 8 to 5 cm H_2_O) was conducted with the dedicated software of the ventilator (Inview PEEP, GE Healthcare). Each PEEP step lasted 8 min, and all other ventilator settings remained unchanged throughout the procedure. At the end of each PEEP step, (1) respiratory mechanics were automatically assessed by the ventilator through 1-s end-inspiratory and end-expiratory holds: plateau pressure and total PEEP were respectively measured at the end of inspiratory and expiratory pauses, and driving pressure (ΔP = plateau pressure – total PEEP) and respiratory system compliance (respiratory system compliance = V_T_/ΔP) were computed by the ventilator; and (2) end-expiratory lung volume was measured by the ventilator (Carescape R860, GE Healthcare) through a modified technique during nitrogen wash-out (20% Fio_2_ increase) and wash-in (20% Fio_2_ decrease), according to a method described elsewhere.^12,15,26^ Wash-out and wash-in data were averaged automatically and considered valid if the difference between the two was less than 20% (cutoff determined by the manufacturer).

At the end of the PEEP trial (*i.e.*, at PEEP 5 cm H_2_O), arterial blood gases were performed, and then a one-breath derecruitment maneuver (5-s exhalation, respiratory rate less than 8 breaths/min) from PEEP 5 cm H_2_O to 0 cm H_2_O was conducted to assess baseline functional residual capacity (FRC), computed as the difference between end-expiratory lung volume at PEEP 5 cm H_2_O and the increase in lung volume over FRC due to the 5 cm H_2_O PEEP. The latter (PEEP5_volume_) was calculated by subtracting the insufflated V_T_ from the expired V_T_ in the first breath after the change in PEEP, with respiratory rate set at 5 breaths/min. This is the way in which PEEP volume can be measured in a noninvasive manner without ventilating the patients at PEEP 0 cm H_2_O, which can be dangerous in patients with ARDS.

PEEP5_volume_ = single-breath exhaled V_T_ – inspired V_T_

FRC = EELV_PEEP5_ – PEEP5_volume_

For each derecruitment step (*i.e.*, from PEEP 15 to 13, 13 to 10, 10 to 8, 8 to 5, and 5 to 0 cm H_2_O), the following parameters were then computed:

#### Alveolar Recruitment and PEEP-induced Inflation^16^.

PEEP-related total volume (PEEPvol) was computed as the difference between end-expiratory lung volume at higher PEEP (EELV_PEEPhigh_) and end-expiratory lung volume at lower PEEP (EELV_PEEPlow_). PEEP-induced inflation volume (*i.e.*, the minimal predicted increase in lung volume due to PEEP) was computed as the product of compliance at lower PEEP (Crs_PEEPlow_) and the PEEP difference between the two steps. PEEP-related recruited volume (V_REC_, ml) was calculated as

V_REC_ = (EELV_PEEPhigh_ – EELV_PEEPlow_) – [(PEEPhigh – PEEPlow) · Crs_PEEPlow_]^14^

Recruited volume was then normalized to the change in PEEP (*i.e.*, compliance of the recruited volume [Crec], ml/cm H_2_O), and the recruitment-to-inflation ratio was calculated as the ratio between Crec and respiratory system compliance at the lower PEEP level, as previously described^16^ (online calculator available at https://crec.coemv.ca/):

Crec = V_REC_/(PEEPhigh – PEEPlow)

Recruitment-to-inflation ratio = Crec/Crs_PEEPlow_

The same parameters were also computed across the whole tested PEEP range by using 15 cm H_2_O as PEEPhigh and 5 cm H_2_O as PEEPlow in all calculations.

#### Strain Indices.

Aerated lung volume (FRC + recruitment) at each PEEP level was calculated as the sum of FRC (*i.e.*, at 0 cm H_2_O PEEP) and the recruited volume above FRC for that PEEP step. Dynamic strain for each PEEP step was computed as V_T_/(FRC + recruitment). Static strain was computed as PEEP-induced inflation volume/(FRC + recruitment): at PEEP = 0 cm H_2_O, static strain is 0. Total strain was calculated as the sum of static and dynamic strain.

After the study end, ventilation was resumed as clinically indicated by the attending physician.

Of note, although physiologic measurements were derived from derecruitment PEEP trials, we refer to the terms “recruitment” and “recruitability” when describing our results. This approach is consistent with what previously done in similar investigations,^6,14,16,27^ aiming at simplifying the interpretation of study results and their clinical implications.

### Endpoints

Study endpoints were (1) to assess the behavior of the recruitment-to-inflation ratio across the traditionally tested 15 to 5 cm H_2_O range and in narrower PEEP ranges; and (2) to establish the relationship between the recruitment-to-inflation ratio and the corresponding PEEP-induced changes in lung dynamic, static, and total strain.

### Sample Size Calculation and Statistical Analysis

Given the physiologic design of the study, consistent with previous investigations with similar design on the topic,^14^ we did not perform a formal sample size calculation. We thus planned to enroll a convenience sample of 20 patients.

Continuous data are expressed as median [interquartile range], and dichotomous variables are displayed as frequencies (%). Paired comparisons between study steps were performed with the Wilcoxon rank sum test: mean differences (95% CI] are displayed for most significant results. Intraindividual variability was rated with the coefficient of variability (ratio of the SD to the mean^28^). Correlations between continuous variables were assessed with Pearson’s correlation test: r and, for significant results, the slope (95% CI] of the linear regression are displayed. The correlation analyses are intended for descriptive purposes only, since the independence assumptions underlying these tests have been violated.

Differences between repeated variables were assessed with repeated ANOVA.

Results with a two-tailed *P* < 0.05 were considered significant. Statistical analysis was performed with SPSS v. 20.0 (SPSS Inc., USA).

### Mathematical Modeling

As an exploratory analysis, recruitment-to-inflation ratio values obtained in each derecruitment step were regressed (by nonlinear ordinary least-squares) against average step PEEP with a quadratic (parabolic) model, constrained to pass through the point (0,0), assuming essentially no recruitment at extremely low PEEP values. A goodness-of-fit test (chi-square) was conducted on each fit. Such an analysis was not prespecified and was included in the analysis during the peer review process.

The rationale of performing such an exploratory analysis by introducing a nonlinear model is that of quantitatively identifying (where possible) the increasing and then decreasing recruitment at progressively increasing PEEP. This behavior is assumed to occur on physiologic principles: at zero end-expiratory pressure, by definition, there is no recruitment; recruitment must tend toward zero at very high PEEP as the maximal capacity of the lung–chest wall is reached on the pressure–volume curve of the respiratory system, corresponding to an ever-decreasing compliance^29^; there should be a maximum recruitment in between. This theoretical nonlinear behavior may, however, be impossible to characterize given the noise in the observations, the limited range explored, the very small size of the sample, and other possible factors. In other words, while the nonlinear model (reputed to represent physiology as we know it) may be *a posteriori* unidentifiable for a number of reasons, we attempt to use it where possible to extract information helpful to the clinician. If for a patient the model proves to be *a posteriori* unidentifiable, then for that patient, the data set is not sufficient for us to draw conclusions.

It should also be appreciated that this approach essentially makes statements about only one (out of possibly many) class of patients, whose data behave in possibly different ways. Indeed, only patients conforming to the increasing and then decreasing pattern are eventually characterized. A formal assessment of what classes actually exist and what (meta)parameters characterize each class (*e.g.*, after a mixed-effects analysis) would require many more individuals: the current approach to identify single patients from just one of such classes is entirely exploratory.

## Results

Twenty-six patients were screened for enrollment. Six patients (23%) showed intrinsic PEEP or airway closure with airway opening pressure greater than 5 cm H_2_O at the screening visit, and were hence excluded. There were no missing, lost, or excluded data from included patients.

Twenty COVID-19 ARDS patients were studied. Demographics and clinical characteristics at enrollment are displayed in Supplementary table 1 (https://links.lww.com/ALN/D257). All patients were studied in semirecumbent position within 24 h of endotracheal intubation, during continuous sedation and paralysis. No changes in any of the experimental settings were needed during the course of the study.

The median Pao_2_/Fio_2_ ratio at enrollment was 107 mmHg [90 to 130]: 12 patients (60%) were classified as having moderate ARDS, and 8 (40%) patients were classified as having severe ARDS. The median FRC was 1,343 ml [953 to 1,682].

Study variables recorded at PEEP 15 cm H_2_O and 5 cm H_2_O are displayed in table 1. Lung volumes and recruitment-derived indices in the narrow PEEP ranges are displayed in table 2.

**Table 1.**
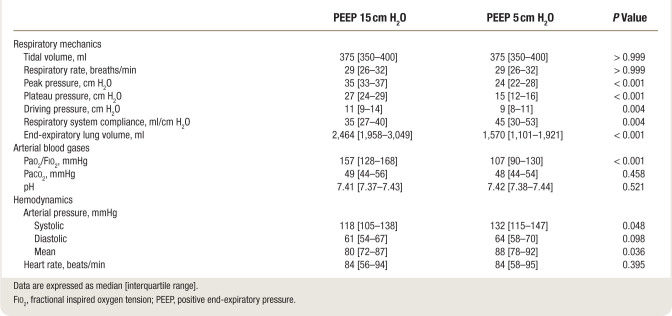
Respiratory Mechanics and Gas Exchange of Enrolled Patients across the Decremental Positive End-expiratory Pressure Trial

**Table 2.**
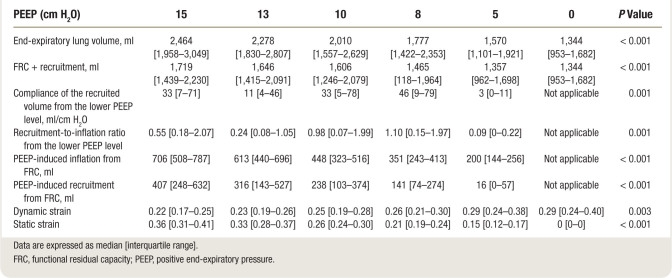
Lung Volumes and Strain Indices Measured in Each Positive End-expiratory Pressure Step

### Potential for Lung Recruitment

Between PEEP 5 and 15 cm H_2_O, median absolute recruited volume was 456 ml [227 to 645], with a Crec of 46 ml/cm H_2_O [23 to 65] and a recruitment-to-inflation ratio of 1.27 [0.40 to 1.69].

**Fig. 2.**
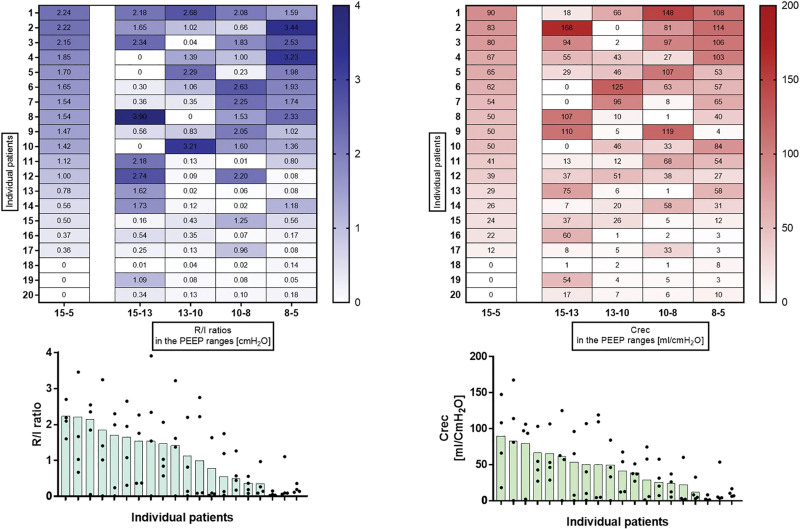
(*Top left*) Heat map showing the recruitment-to-inflation ratio from 15 to 5 cm H_2_O (*first column*) and the recruitment-to-inflation ratio measured within the narrower positive end-expiratory pressure (PEEP) ranges of the trial. Each *row* represents an individual patient. (*Top right*) Heat map showing the compliance of the recruited volume (ml/cm H_2_O) from 15 to 5 cm H_2_O (*first column*) and the compliance of the recruited volume measured within the narrower PEEP ranges of the trial. Each row represents an individual patient. *Darker colors* indicate higher values of recruitment-to-inflation ratio and compliance of the recruited volume, respectively. Individual patients are ordered according to the 15– 5–cm H_2_O recruitment-to-inflation ratio values, from highest to lowest (and hence from the *darkest to the lightest color* in the *first column*). (*Bottom*) Intrapatient variability between the recruitment-to-inflation ratio (*left*) and compliance of the recruited volume (*right*) in narrow PEEP ranges. Each *bar* represents the value obtained across the 10–cm H_2_O range. *Black dots* represent the four values obtained in the PEEP steps. Each column represents an individual patient.

Crec and recruitment-to-inflation ratio in the four PEEP ranges along with global Crec and recruitment-to-inflation ratio measured between PEEP 15 and 5 cm H_2_O are displayed in figure 2.

**Fig. 3.**
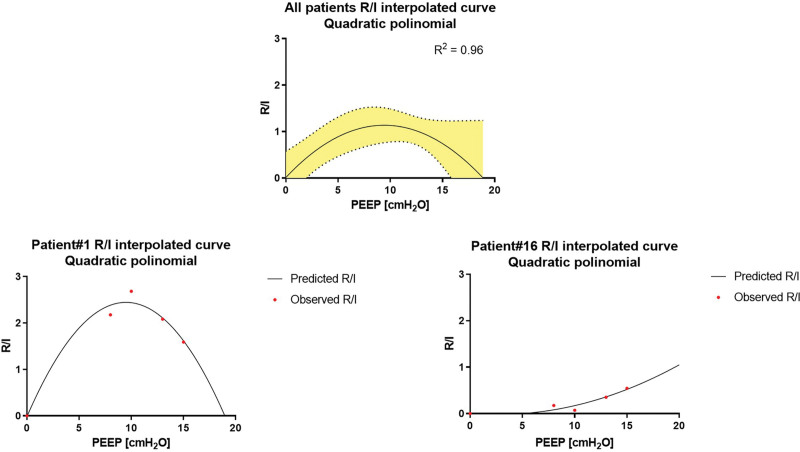
(*Top*) Nonlinear fit of the recruitment-to-inflation ratio *versus* positive end-expiratory pressure (PEEP) over the decremental steps yielded a quadratic (parabolic) curve for average values from all studied patients (mean and standard error displayed). (*Bottom*) Nonlinear fit of the recruitment-to-inflation ratio *versus* PEEP in two representative patients. This figure shows the behavior of two typical patients, No. 1 (*left*, with a saturating recruitment-to-inflation ratio response to increasing PEEP) and No. 16 (*right*, with an ever-increasing recruitment-to-inflation ratio response to increasing PEEP).

Average recruitment-to-inflation ratio measured in the four narrow PEEP ranges (median, 1.11 [0.38 to 1.52]) was highly correlated (r = 0.92) with the recruitment-to-inflation ratio measured between PEEP 15 and 5 cm H_2_O; similarly, average Crec measured in the four narrow PEEP ranges (median 39 ml/cm H_2_O [17 to 59]) highly correlated with Crec measured between PEEP 15 and 5 cm H_2_O (r = 0.96). However, both Crec and recruitment-to-inflation ratio had great intraindividual variability in the four PEEP ranges: median intraindividual Crec and recruitment-to-inflation ratio coefficient of variability were 81% [65 to 114] and 85% [70 to 109], respectively (fig. 2). Specifically, nonlinear fit of recruitment-to-inflation ratio *versus* PEEP over the decremental PEEP steps yielded a quadratic (parabolic) curve for 17 of the 20 studied patients (fitting with parabolic polynomial model *R*^2^ = 0.96). For three patients (No. 8, 11, and 14), the model was significantly incorrect and could not be used for predictions (fig. 3, Supplemental table 2, https://links.lww.com/ALN/D257). Clearly, the fit is, in several instances, very problematic: we indicate with the value “greater than 20” doubtful results, indicating extreme, possibly implausible estimates. For this reason, only patient datasets for whom the model fit passes the goodness-of-fit test should be considered to provide useful information.

### Respiratory Mechanics and Lung Strain

As compared to PEEP 5 cm H_2_O, PEEP 15 cm H_2_O yielded reduced respiratory system compliance (mean difference, 9 ml/cm H_2_O [95% CI, –13 to –3]) and increased driving pressure (mean difference, 2 cm H_2_O [95% CI, 1 to 3]).

As expected, dynamic strain at PEEP 15 cm H_2_O was lower than dynamic strain at PEEP 5 cm H_2_O (mean difference, –0.09 [95% CI, –0.13 to –0.05]); conversely, static strain was higher at PEEP 15 than at PEEP 5 cm H_2_O (mean difference, 0.08 [95% CI, 0.04 to 0.12]).

### Lung Strain Indices

Respiratory system compliance and aerated lung volume (FRC + recruitment) were linearly correlated at PEEP 5 cm H_2_O (r = 0.83), while the relationship became weaker at PEEP 15 cm H_2_O (r = 0.52). Correspondingly, the driving pressure was linearly correlated to dynamic strain at PEEP 5 cm H_2_O (r = 0.79), but not at PEEP 15 cm H_2_O (r = 0.22; Supplementary figure 1, https://links.lww.com/ALN/D255).

**Fig. 4.**
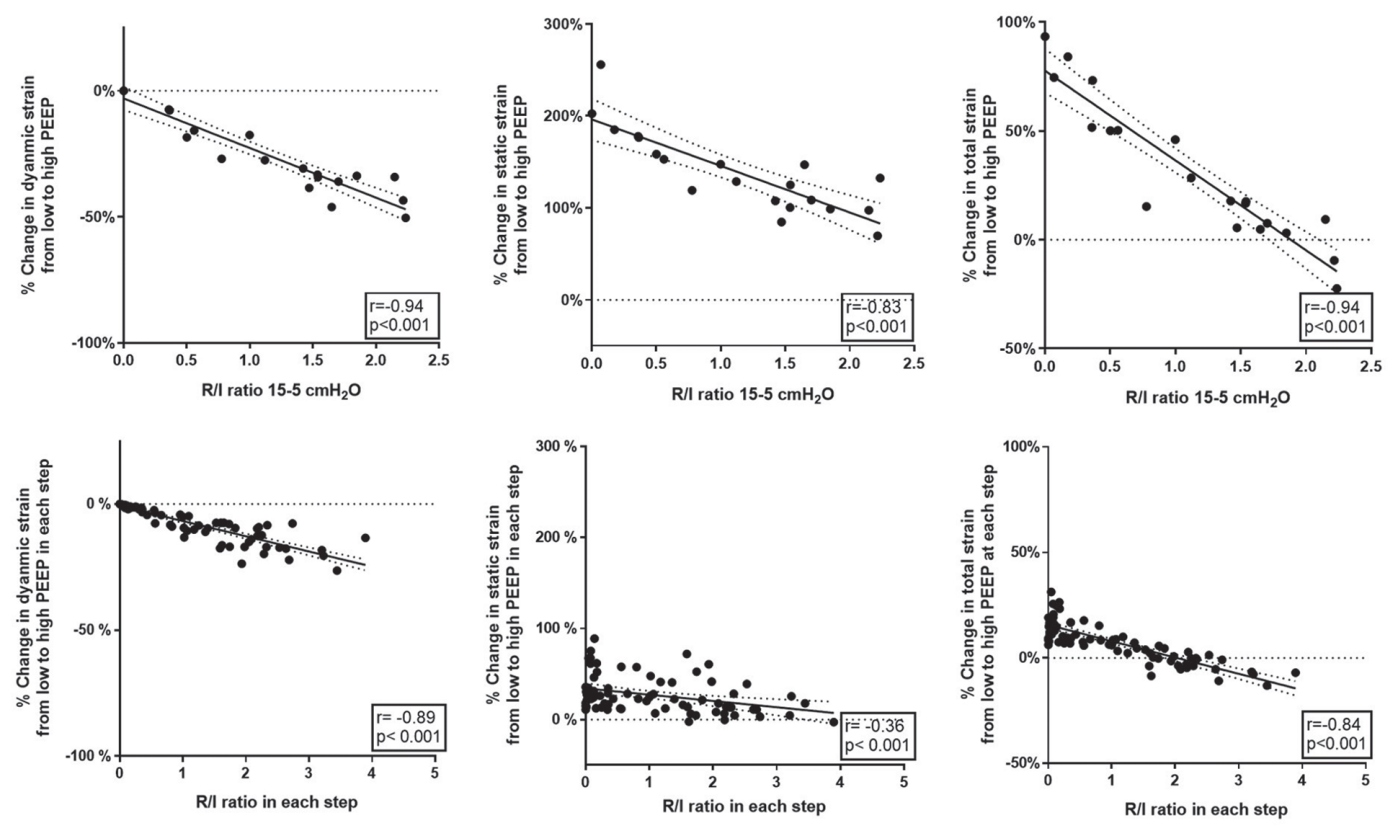
Relationship between the PEEP-induced percent change in dynamic strain, static strain, and total strain, and the corresponding recruitment-to-inflation ratio measured between 5 and 15 cm H_2_O of positive end-expiratory pressure (PEEP; *upper row*) and within narrower PEEP ranges (*lower row*). Recruitment-to-inflation ratio, both measured across a 10–cm H_2_O range and within narrower PEEP ranges, has a strong relationship with the reduction in lung strain induced by PEEP. Specifically, patients with higher recruitment-to-inflation ratio exhibit a greater reduction in dynamic strain and a lower increase in static strain due to PEEP.

The reduction dynamic and total strain and the increase in static strain produced by the 10–cm H_2_O PEEP increase had reverse linear correlation with the corresponding recruitment-to-inflation ratio (dynamic strain, r = –0.94; static strain, r = –0.83; total strain, r = –0.94; fig. 4, *top*).

Also, reduction in dynamic and total strain and the increase in static strain produced by narrower PEEP levels tested in the decremental PEEP trial were closely related to the corresponding recruitment-to-inflation ratio measured (dynamic strain, r = –0.89; static strain, r = –0.36; total strain, r = –0.84; fig. 4, *bottom*).

Changes in respiratory system compliance, driving pressure, and Pao_2_/Fio_2_ produced by the 10–cm H_2_O PEEP increase were not related to the corresponding Crec or to PEEP-induced changes in static, dynamic, and total lung strain (Supplementary figure 2, https://links.lww.com/ALN/D256).

## Discussion

The results of the current physiologic study conducted on patients with early moderate-to-severe COVID-19 ARDS can be summarized as follows:

1. Recruitment-to-inflation ratio calculated between 15 and 5 cm H_2_O represents average recruitment in that range. However, this corresponds to a nonuniform behavior of recruitment: in most cases (85%), the relationship between recruitment-to-inflation ratio and PEEP fits a quadratic (parabolic) curve.
2. Respiratory mechanics changes induced by PEEP are ineffective estimates of the changes produced on lung strain (Supplementary figure 2, https://links.lww.com/ALN/D256). This occurs because the proportionalities between aerated volume *versus* respiratory system compliance and dynamic strain *versus* driving pressure become weaker as high PEEP is applied.
3. Recruitment-to-inflation ratio, calculated both between PEEP 15 to 5 cm H_2_O and within narrower ranges through end-expiratory lung volume measurement during a five-step decremental PEEP trial, is proportional to PEEP-induced reduction in dynamic and total lung strain. Its use at the bedside to mechanistically titrate PEEP appears physiologically sound.
4. Because of the nonuniform behavior of recruitment-to-inflation ratio *versus* PEEP across the 15– to 5–cm H_2_O PEEP range, sequential lung volume measurement with recruitment-to-inflation ratio calculation within narrower ranges during a decremental PEEP trial allows more granular estimation of PEEP effects on lung strain. Clinically, this may be particularly relevant to aid PEEP titration in patients exhibiting intermediate recruitment-to-inflation ratio values in the simplified maneuver across the conventionally tested 15– to 5–cm H_2_O PEEP range.

In ARDS, it is widely accepted that PEEP setting should aim at a balance between alveolar recruitment (*i.e.*, reopening of alveolar units yielding increased lung volume available for tidal ventilation) and the damage unavoidably generated in the already open tissue. During recent decades, great effort has been made to identify the PEEP-setting strategies to balance the need for lung recruitment and limiting PEEP-induced alveolar overdistension; PEEP titration methods based on respiratory system compliance, oxygenation, shunt values, and pressure–volume curve have been proposed. Five different randomized studies comparing higher *versus* lower PEEP, with high PEEP set according to respiratory system mechanics,^30^ according to oxygenation impairment,^31,32^ to maximize respiratory system compliance,^33^ or to achieve different degrees of positive transpulmonary pressure,^34^ failed to detect a clinical benefit.

A drawback of “universal” strategies driving the setting of high or low PEEP is that the potential for lung recruitment has great interindividual variability, with high PEEP enhancing lung injury in patients with low recruitability, and low PEEP not fully exerting its beneficial effects in recruitable patients.^4,5,11,16,35,36^ Thus, a search for a tool to individualize PEEP based on a patient’s individual response appears relevant.

The recently developed recruitment-to-inflation ratio may offer a simple, timely, and reproducible assessment of recruitability at the bedside.^16^ The advantages of this method include its simplicity of use and no need for additional equipment: these features make this approach feasible even in limited-resources settings.^17,37–40^ Assessment of the recruitment-to-inflation ratio is based on a simplified technique that can be applied with all ICU ventilators, as it requires only an abrupt change in PEEP with the ability to monitor exhaled tidal volume. The recruitment-to-inflation ratio technique itself has limitations: errors in measurements of relevant variables have been shown with several ICU ventilators,^19^ and careful equipment checks are warranted for appropriate application. An additional drawback of the technique is that of providing the potential for lung recruitment across a wide PEEP range (15 to 5 cm H_2_O, in most cases). It is unknown if recruitability is uniform in a 10–cm H_2_O PEEP range. A recruitment-to-inflation ratio threshold of 0.5 has been suggested to separate patients having high or low potential for lung recruitment^16^: this may yield a dichotomous definition of patients as having high or low potential for lung recruitment (*i.e.*, benefiting or not from high PEEP). Importantly, recruitment-to-inflation ratio values close to 0.5 are commonly observed in clinical practice,^6,16,17,40^ and these may be difficult to interpret to properly choose between a higher *versus* lower PEEP strategy.^19^ Moreover, whether the recruitment-to-inflation ratio accurately reflects the changes in dynamic strain produced by PEEP is yet to be demonstrated: this appears to be of paramount importance, as PEEP prevents ventilator-induced lung injury only if it yields sufficient recruitment to reduce the dynamic strain caused by tidal volume in the aerated lung.^20^

Our study demonstrates the reliability of the recruitment-to-inflation ratio measured between PEEP 15 and PEEP 5 cm H_2_O to assess bedside the effects of PEEP on lung strain. Importantly, we show that PEEP-induced effects on lung strain have no correlation with changes in compliance, driving pressure, or oxygenation as a response to PEEP (Supplementary figure 2, https://links.lww.com/ALN/D256), which indicates that these parameters may not represent sensitive tools to estimate recruitability.^33,41–43^

High recruitment-to-inflation ratio values (*e.g.*, above 1) identify patients in whom PEEP efficiently reduces dynamic and total strain, with minimal increases in static strain. Low recruitment-to-inflation ratio (*e.g.*, next to 0) values identify patients in whom PEEP only induces increases in static strain with minimal or no reduction in dynamic and total strain. In contrast, intermediate recruitment-to-inflation ratio values (*i.e.*, close to 0.5 to 0.7) are difficult to interpret to properly titrate PEEP^19^: the findings of our study indicate that these may reflect a nonuniform behavior of the potential for lung recruitment across the conventionally tested 10–cm H_2_O PEEP range during the simplified maneuver. In this latter population, sequential end-expiratory lung volume–guided assessment of the recruitment-to-inflation ratio within narrower PEEP ranges during a decremental PEEP trial may allow precise detection of the changes in dynamic and total strain produced by PEEP. This is a simple automated procedure that is available at the bedside and requires specific equipment integrated in the mechanical ventilator, but no additional invasiveness.

The further modeling analysis presented in our manuscript showed a nonlinear fit of the recruitment-to-inflation ratio *versus* PEEP over the decremental steps, which yielded a quadratic (parabolic) curve for 17 of 20 patients. Granular recruitment-to-inflation ratio values obtained across decreasing PEEP steps may aid clinical choice of the proper PEEP level in two possible ways: on the one hand, if the model reliably captures the recruitment-to-inflation ratio trend over increasing PEEP, much of the observation noise is averaged out, and a credible prediction on the effective level of PEEP may be made; on the other hand, the goodness-of-fit test objectively excludes those patients in whom the procedure delivers unreliable results: faced either with a significantly noncorrect model or with effective PEEP beyond the usual operational range, the clinician can decide either to follow traditional criteria and assign the PEEP disregarding the results of the derecruitment procedure, or to repeat the derecruitment procedure, possibly after appropriate therapy adjustments (position, aspiration, check for leaks). It should, however, be underscored that the use of individual, single-patient model estimation (without any attempt at estimating group metaparameters such as in a mixed-effects approach) leads to a very large number of degrees of freedom, with few points being available to estimate the necessary parameters, with a substantial risk of overfitting. On the other hand, we feel the procedure is justified in that the form of the model is constrained by commonly agreed upon physiologic considerations, thus allowing the *a priori* exclusion of whole families of submodels (*e.g.*, linear).

Our study may have important clinical implications:

1. Dynamic strain is the key mechanism of lung injury during ARDS.^7,44–48^ Our study demonstrates that the recruitment-to-inflation ratio accurately reflects the effects of PEEP on lung strain. Its use to mechanistically titrate PEEP at the bedside appears promising in terms of physiologic soundness.
2. The recruitment-to-inflation ratio measured in the 15– to 5–cm H_2_O PEEP range represents the average recruitability in that range; however, its behavior is not linear but parabolic in most patients. This is particularly relevant in patients exhibiting the recruitment-to-inflation ratio assessed across the conventionally tested 10–cm H_2_O PEEP range within intermediate values, which are difficult to interpret to properly choose between a lower *versus* higher PEEP strategy.
3. In selected patients (those with an intermediate recruitment-to-inflation ratio), sequential end-expiratory lung volume measurement enabling recruitment-to-inflation ratio calculation within narrower ranges during a decremental PEEP trial may enable more granular evaluation of PEEP effects on lung strain. This may aid more precise bedside PEEP titration within the evaluated range, setting PEEP to exploit lung recruitment when it is present while minimizing lung hyperinflation when recruitment does not occur.

Our study has limitations:

1. In our study, the recruitment-to-inflation ratio was calculated from the lung volume change due to PEEP measured with the dilution technique, and not estimated by the simplified derecruitment maneuver.^16^ Since with the simplified derecruitment maneuver the difference between the single-breath exhaled tidal volume after PEEP lowering and set tidal volume represents the PEEP-induced change in lung volume, the latter may be underestimated. This is the reason recruitment-to-inflation ratio values in our study are higher than those reported in previous investigations. The dilution technique represents the reference method for assessing lung volumes^14,49^: this does not alter, and could even strengthen, the meaning of this investigation. However, these considerations prompt caution regarding the interpretation of absolute recruitment-to-inflation ratio values displayed in our study, as these cannot be compared to those obtained with the simplified derecruitment maneuver.
2. Patients with airway closure or intrinsic PEEP were excluded from the study: this has helped avoid further confounding factors that could have required even more complex data analysis and interpretation for this proof-of-concept study.
3. We refer to the terms “recruitment” and “recruitability,” although our results should be more properly referred to as “derecruitment” data. This conceptual shift may represent a limitation, considering the hysteretic behavior of pressure–volume curves. Nonetheless, this approach is consistent with the most recent literature on the topic, as derecruitment is simpler to assess and not significantly different from recruitment when a prolonged high-PEEP stabilizing phase precedes the derecruitment procedure, as in our study.^6,14–16^
4. Due to the pandemic surge, all studied patients suffered from ARDS caused by COVID-19. There are no reasons to hypothesize that our approach based on end-expiratory lung volume–guided recruitment-to-inflation ratio assessment within narrow PEEP ranges should not be applicable to ARDS from other causes, since respiratory mechanics in COVID-19 ARDS have been shown to share a high degree of similarity with non–COVID-19 cases.^6,36,50^
5. No biologic or imaging techniques were used to support the findings of our study. The latter represents a limitation both due to of the lack of data on the regional distribution of ventilation and because computed tomography scan validation of recruitment evaluation is not present. Nevertheless, the role of dynamic strain as key determinant of ventilator-induced lung injury is well-established,^20^ and these limitations are intrinsic to the recruitment-to-inflation ratio technique, as previously pointed out. Moreover, the dilution technique represents the accepted standard for lung volume determination and has been validated against pressure–volume curves for the study of alveolar recruitment.^14^ We believe that this choice increases the physiologic reliability of our data even in the absence of imaging techniques.
6. Caution is warranted when interpreting linear correlation data from our study: indeed, due to the lack of an external validation of recruitment such as imaging techniques, correlations between recruitment-to-inflation ratio and the recruitment index Crec/FRC and between recruitment-to-inflation ratio and changes in strain must take into account a possible degree of bias, since a number of parameters are involved in the computation of both elements of the correlation.

### Conclusions

Recruitment-to-inflation ratio assessed by reducing PEEP from 15 to 5 cm H_2_O accurately estimates PEEP-induced reduction in lung strain. This can be measured through a simplified maneuver that can be done with any ICU ventilator: its bedside use to choose between a higher *versus* lower high PEEP strategy appears promising. However, recruitability may not be uniform between PEEP 5 and 15 cm H_2_O, with most patients showing a parabolic behavior of recruitment-to-inflation ratio across this range. Intermediate recruitment-to-inflation ratio values (*e.g.*, slightly above or below the proposed cutoff of 0.5 to 0.7) across the conventional 15– to 5–cm H_2_O PEEP range are commonly observed in clinical practice and difficult to interpret. In these cases, end-expiratory lung volume measurement with recruitment-to-inflation ratio calculation within narrower ranges during a decremental PEEP trial enables more granular assessment of recruitability and PEEP effects on lung strain.

### Research Support

Support was provided solely from institutional and/or departmental sources.

### Competing Interests

Dr. Grieco has received payments for travel expenses from Getinge (Göteborg, Sweden), and personal fees from GE Healthcare (Chicago, Illinois), Intersurgical (Mirandola, Italy), Fisher & Paykel Healthcare (Auckland, New Zealand), and Merck Sharp & Dohme (Rahway, New Jersey). Dr. Maggiore is the principal investigator of the Re-intubation after nasal high-flow (RINO) trial (www.clinicaltrials.gov, NCT02107183), which was supported by Fisher & Paykel Healthcare, and declares receiving speaker’s fees from GE Healthcare, Masimo (Irvine, California), Aspen (Durban, South Africa), and Getinge and participating in an advisory board for Sanofi (Paris, France). Dr. Antonelli has received payments for board participation from Maquet (Rastatt, Germany), Air Liquide (Paris, France), and Chiesi (Parma, Italy). Drs. Grieco and Antonelli disclose a research grant from GE Healthcare. The other authors declare no competing interests.

## Supplemental Digital Content

Supplementary Figure 1: Relationship between aerated lung size and respiratory system compliance and dynamic strain and driving pressure measured at low (*left*) and high positive end-expiratory pressure (*right*). https://links.lww.com/ALN/D255

Supplementary Figure 2: Respiratory system compliance, driving pressure, and Pao_2_/fractional inspired oxygen tension are unreliable estimates of positive end-expiratory pressure–induced alveolar recruitment and changes in lung strain. https://links.lww.com/ALN/D256

Supplementary Table 1: Demographics and baseline characteristics of enrolled patients. https://links.lww.com/ALN/D257

Supplementary Table 2: Nonlinear fit of the relationship between recruitment-to-inflation ratio and positive end-expiratory pressure. https://links.lww.com/ALN/D257

## Supplementary Material
