# Supplementary material for: Recruitment-to-inflation Ratio Assessed through Sequential End-expiratory Lung Volume Measurement in Acute Respiratory Distress Syndrome
Source: Anesthesiology. 2023 Jul 31;139(6):801–14. doi: 10.1097/ALN.0000000000004716 (PMC10723770; doi:10.1097/ALN.0000000000004716)
Supplement: Supplementary file 1 [file aln-139-801-s001.pdf]

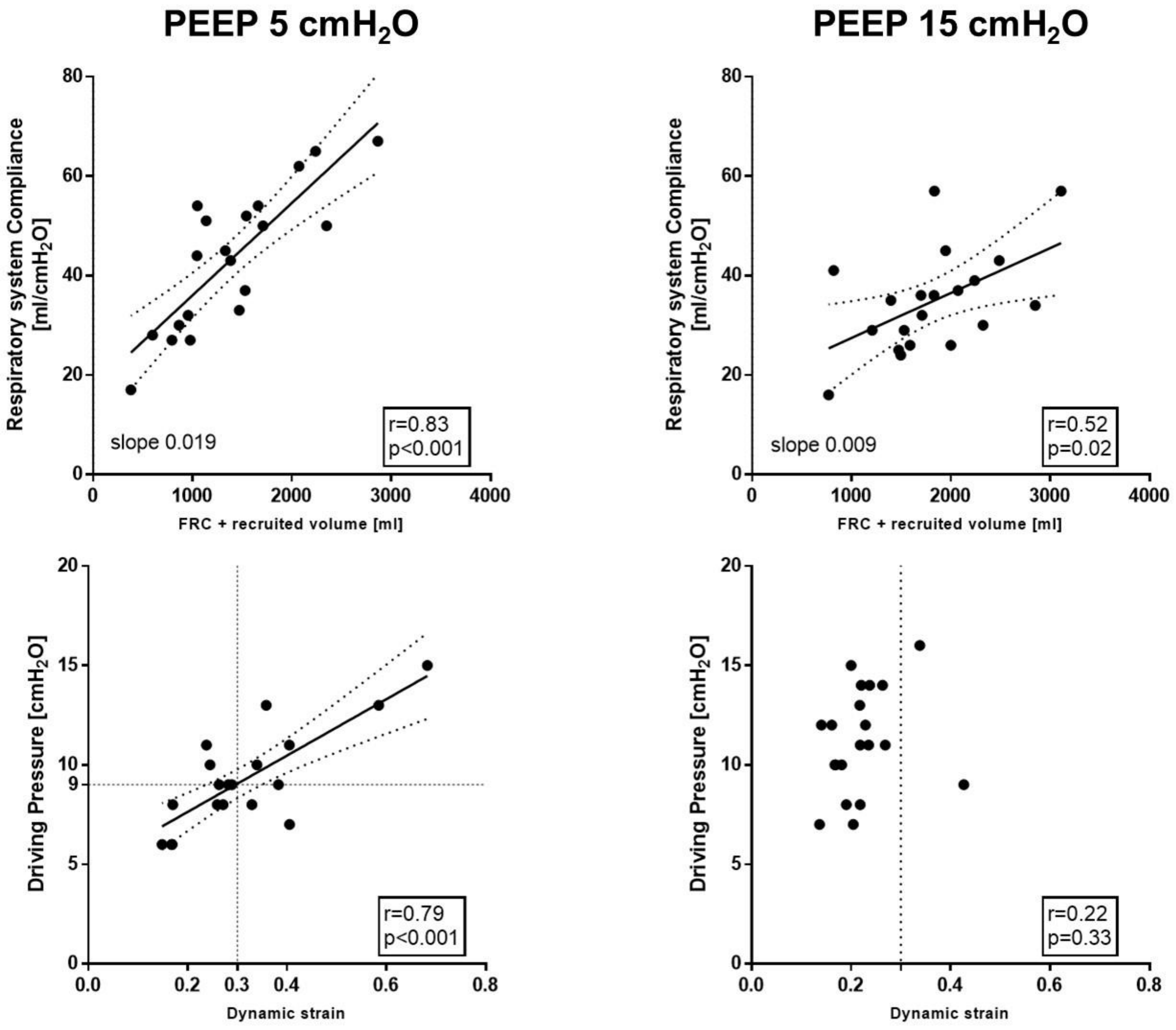

Supplementary Figure 1

Relationship between aerated lung size/respiratory system compliance and dynamic strain/driving pressure measured at low (left) and high PEEP (right).
