# Supplementary material for: Recruitment-to-inflation Ratio Assessed through Sequential End-expiratory Lung Volume Measurement in Acute Respiratory Distress Syndrome
Source: Anesthesiology. 2023 Jul 31;139(6):801–14. doi: 10.1097/ALN.0000000000004716 (PMC10723770; doi:10.1097/ALN.0000000000004716)
Supplement: Supplementary file 2 [file aln-139-801-s002.pdf]

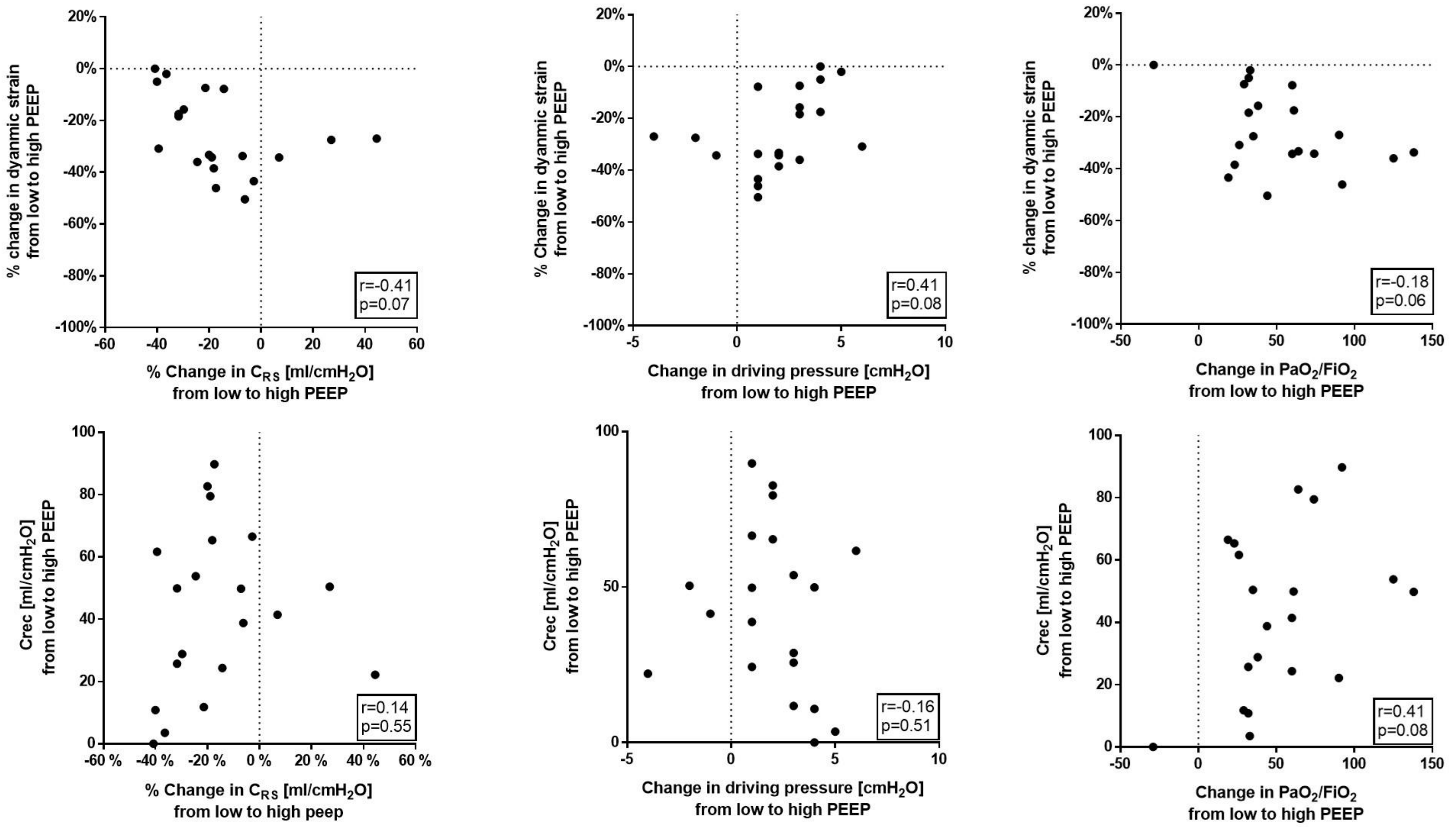

Supplementary Figure 2

Respiratory system compliance (Crs), driving pressure and PaO_2/FiO_2 are unreliable estimates of PEEP-induced alveolar recruitment and changes in lung strain.

Top: relationship between the changes in dynamic, static and total strain induced by PEEP, and the corresponding changes in respiratory system compliance (Crs), driving pressure and PaO_2/FiO_2.

Bottom: relationship between the amount of alveolar recruitment (Crec) induced by PEEP, and the corresponding changes in respiratory system compliance (Crs), driving pressure and PaO_2/FiO_2.
