# Supplementary material for: Recruitment-to-inflation Ratio Assessed through Sequential End-expiratory Lung Volume Measurement in Acute Respiratory Distress Syndrome
Source: Anesthesiology. 2023 Jul 31;139(6):801–14. doi: 10.1097/ALN.0000000000004716 (PMC10723770; doi:10.1097/ALN.0000000000004716)
Supplement: Supplementary file 3 [file aln-139-801-s003.pdf]

| **Supplementary Table 1.** Demographics and baseline characteristics of enrolled patients | | |
|---|---|---|
| Age, years | | 63 [56-74] |
| Female sex | no. (%) | 2 (10) |
| Height, cm | | 175 [171-180] |
| Predicted body weight, kg | | 70 [69-75] |
| Body mass index, kg/m$^2$ | | 29 [26-32] |
| SOFA at study inclusion | | 4 [3-5] |
| SAPS II | | 37 [24-56] |
| *Comorbidities* | no. (%) | |
| Hypertension | | 10 (50) |
| Active cancer | | 1 (5) |
| Chronic obstructive pulmonary disease | | 1 (5) |
| Diabetes mellitus | | 5 (25) |
| Coronary artery disease | | 1 (5) |
| Noninvasive respiratory support before intubation | no. (%) | 10 (50) |
| Duration of noninvasive respiratory support before intubation, hours | | 59 [20-72] |
| ARDS severity at enrollment | no. (%) | |
| Moderate ($PaO_2/FiO_2$ ratio 101-200 mmHg) | | 12 (60) |
| Severe ($PaO_2/FiO_2$ ratio ≤100 mmHg) | | 8 (40) |
| *Clinical outcome* | | |
| Prone positioning during the ICU stay | no. (%) | 20 (100) |
| Acute kidney failure | no. (%) | 7 (35) |
| ICU-acquired infection | no. (%) | 19 (95) |
| Pneumothorax | no. (%) | 8 (40) |
| Tracheostomy | no. (%) | 7 (35) |
| 28-day outcome | no. (%) | |
| Dead | | 8 (40) |
| Alive, receiving mechanical ventilation | | 6 (30) |
| Alive, breathing unassisted | | 6 (30) |
| In-ICU mortality | | 9 (45) |
| Data expressed in median [interquartile range], if not otherwise specified. | | |

**Supplementary table 2. Nonlinear fit of the relationship between R/I vs. PEEP.**

For each patient the linear and quadratic regression coefficients are reported, together with the predicted PEEP level at which R/I decreases below 1.0 or 0.5: values of PEEP “ > 20” indicate an ever-increasing recruitment with increasing PEEP. In the last column, the P-value corresponding to the Chi-square goodness-of-fit test is reported.

The table reports, for every patient (first column) the coefficients of the linear (second column) and of the quadratic (third column) terms of the nonlinear model. The PEEP levels at which R/I decreases below 0.5 (fourth column) or 1 (fifth column), where computable, are reported. The significance of a Chi-Square Goodness-of-Fit test on the model is reported in the sixth column: for subjects 8, 11 and 14 the test is significant, and the model must be considered inappropriate in representing the observations.

| subject | b1 | b2 | PEEP@RI=0.5 | PEEP@RI=1 | P2DF |
|---|---|---|---|---|---|
| 1 | 0.36 | -0.01 | > 20 | > 20 | 0.770 |
| 2 | 0.47 | -0.03 | 15.41 | 14.06 | 0.093 |
| 3 | 0.37 | -0.02 | 17.89 | 16.10 | 0.096 |
| 4 | 0.67 | -0.05 | 12.97 | 12.06 | 0.373 |
| 5 | 0.41 | -0.03 | 13.65 | 11.94 | 0.091 |
| 6 | 0.62 | -0.04 | 13.50 | 12.51 | 0.585 |
| 7 | 0.52 | -0.04 | 13.08 | 11.81 | 0.155 |
| 8 | 0.13 | 0.00 | > 20 | > 20 | 0.042 |
| 9 | 0.20 | -0.01 | 19.39 | 14.49 | 0.269 |
| 10 | 0.51 | -0.03 | 14.71 | 13.48 | 0.101 |
| 11 | -0.10 | 0.02 | > 20 | > 20 | 0.000 |
| 12 | -0.01 | 0.01 | > 20 | > 20 | 0.056 |
| 13 | -0.15 | 0.02 | > 20 | > 20 | 0.675 |
| 14 | 0.01 | 0.01 | > 20 | > 20 | 0.043 |
| 15 | 0.24 | -0.02 | 12.16 | | 0.545 |
| 16 | -0.01 | 0.00 | > 20 | > 20 | 0.664 |
| 17 | 0.11 | -0.01 | | | 0.303 |
| 18 | 0.02 | 0.00 | | | 0.948 |
| 19 | 0.00 | 0.00 | > 20 | > 20 | 0.379 |
| 20 | 0.01 | 0.00 | > 20 | > 20 | 0.705 |
